# Reactivation of Pulmonary Tuberculosis following Treatment of Myelofibrosis with Ruxolitinib

**DOI:** 10.1155/2016/2389038

**Published:** 2016-10-23

**Authors:** Maheen Z. Abidi, Javeria Haque, Parvathi Varma, Horatiu Olteanu, Guru Subramanian Guru Murthy, Binod Dhakal, Parameswaran Hari

**Affiliations:** ^1^Division of Infectious Diseases, Department of Medicine, University of Colorado, Denver, CO, USA; ^2^Division of Infectious Diseases, Department of Medicine, Medical College of Wisconsin, Milwaukee, WI, USA; ^3^University of Birmingham Medical School, Edgbaston, Birmingham, UK; ^4^Department of Pathology, Medical College of Wisconsin, Milwaukee, WI, USA; ^5^Division of Hematology and Oncology, Department of Medicine, Medical College of Wisconsin, Milwaukee, WI, USA

## Abstract

Ruxolitinib is widely in use for treatment of myeloproliferative disorders. It causes inhibition of the Janus kinase (JAK) signal transducer and activation of transcription (STAT) pathway, which plays a key role in the underlying pathophysiology of myeloproliferative diseases. We describe a case of reactivation pulmonary tuberculosis in a retired physician while on treatment with ruxolitinib. We also review the literature on opportunistic infections following use of ruxolitinib. Our case highlights the importance of screening for latent tuberculosis in patients from highly endemic areas prior to start of therapy with ruxolitinib.

## 1. Introduction

Janus kinase (JAK) inhibitors, such as ruxolitinib(INCB018424), are being widely used for their excellent efficacy in decreasing the constitutional symptoms and splenomegaly in patients with myeloproliferative neoplasms such as primary myelofibrosis (MF) [1]. The JAK-STAT (signal inducer and activator of transcription) pathway is essential for host immunity and defense [2, 3]. Clinical trials of ruxolitinib have not shown a significant increase in infectious complications [4]. However, several case reports have been published recently describing opportunistic infections in patients on treatment with ruxolitinib [5–15]. We report a case of reactivation pulmonary tuberculosis (TB) following ruxolitinib therapy.

## 2. Case

A 69-year-old male, retired physician who practiced in India was diagnosed with primary myelofibrosis in May 2015. His initial presentation included anemia with massive splenomegaly. Spleen size was evaluated by USS and measured 28 cm in long axis. Constitutional symptoms at diagnosis included night sweats, abdominal pain, weight loss, itching, fatigue, and early satiety. His medical history was negative for major infectious disease. Bone marrow biopsy confirmed MF, grade MF 3 [16], JAK2V617F mutation negative, MPL exon 10 mutation negative, and CALR mutation positive. This was a type 1 mutation with 52 bp deletion in exon 9 of CALR gene.

**Figure 1:**
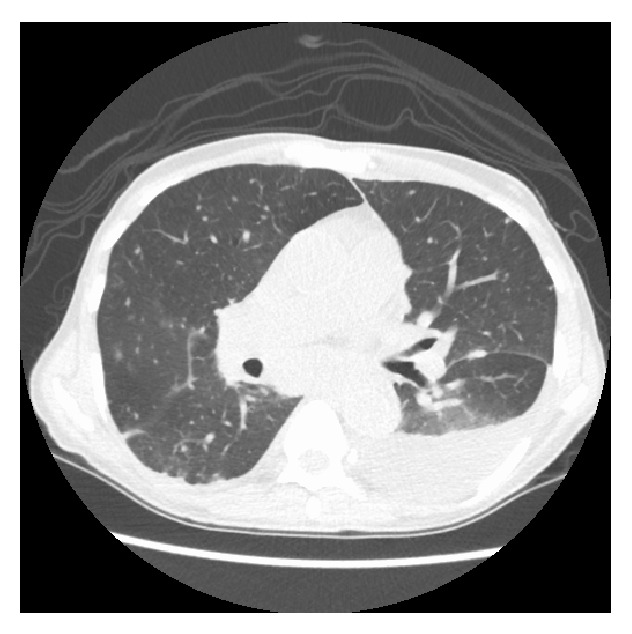
CT scan of chest showing diffuse lung nodules bilaterally, left sided effusion, and mediastinal adenopathy.

**Figure 2:**
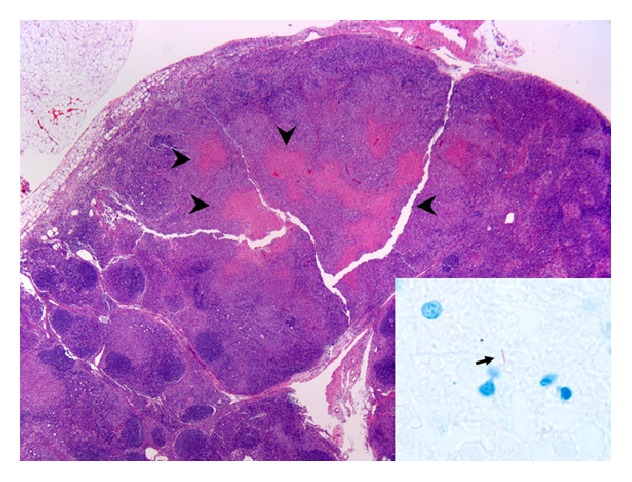
Supraclavicular lymph node (hematoxylin and eosin stain, 20x) with partially effaced architecture and necrotizing granulomatous inflammation (arrow heads). Focal extramedullary hematopoiesis was also present. The arrow in the inset indicates an acid-fast organism (AFB stain, 1,000x).

**Figure 3:**
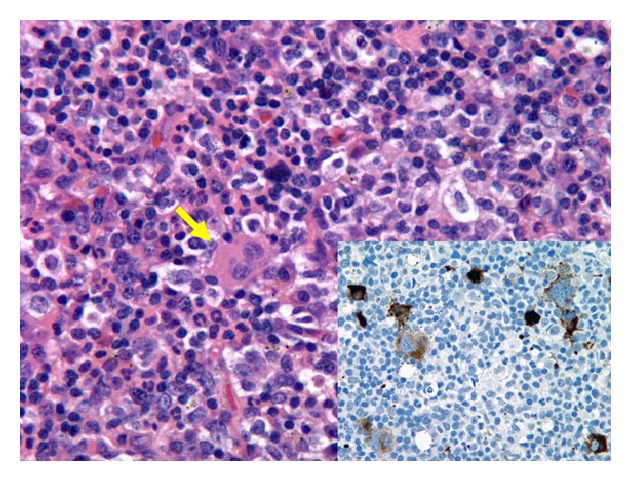
Supraclavicular lymph node (hematoxylin and eosin stain, 500x) with focal areas of extramedullary hematopoiesis, including megakaryocytes (yellow arrow). The inset (CD61 immunohistochemistry, 500x) highlights frequent atypical megakaryocytes (brown) staining positive for CD61 (a platelet and megakaryocytes marker), consistent with the underlying diagnosis of primary myelofibrosis.

He had an intermediate 2 DIPSS plus score and was started on ruxolitinib at 20 mg twice daily for symptom relief. He had a rapid improvement in his constitutional symptoms in the first three weeks of treatment with ruxolitinib. Prior to receipt of ruxolitinib, a screening chest X-ray was negative. Three weeks after initiation of ruxolitinib therapy, he was admitted to the hospital with high-grade fevers (*T*_max_ 102°F), night sweats, shortness of breath, and nonproductive cough. His physical exam revealed matted cervical lymphadenopathy and splenomegaly. QuantiFERON-TB test (Celestis, Valencia, CA) was positive. Computed tomography (CT) of the chest showed bilateral lung nodules, left sided pleural effusion, and lower cervical and mediastinal conglomerate adenopathy (Figure 1). An excisional lymph node biopsy of a cervical node showed necrotizing granulomatous inflammation and rare acid-fast bacilli (AFB) (see Figures 2 and 3). Lymph node tissue cultures were positive for* Mycobacterium tuberculosis *complex by gene-probe (GenProbe, San Diego, CA). Ruxolitinib was discontinued and standard 9-month four-drug antituberculosis therapy (ATT) with rifampin, isoniazid, pyrimethamine, and ethambutol was started which led to rapid improvement in his symptoms. After 6 months of successful ATT, ruxolitinib was reintroduced for his MF symptoms, and he was continued on ATT with isoniazid and rifampin. At follow-up, he remains without transfusion needs and is symptomatically improved with minimal constitutional symptoms. There is complete resolution of lung nodules.

## 3. Discussion

The JAK-signal transducer and activator of transcription (STAT) pathway plays a critical role in host defenses and cell mediated immunity. Use of ruxolitinib in patients with myelofibrosis can cause inhibition of the JAK-STAT signaling, thus leading to depressed T helper cell type 1 response and a reduction of multiple cytokine production including IFN-*γ* and TNF-*α*. T-Inflammatory cytokines, interferon-gamma (IFN-*γ*), and tumor necrosis factor-*α* (TNF-*α*) have a critical role in prevention of reactivation and control of TB infection [6, 9]. TNF-*α* plays a crucial role in T cell function, macrophage activation, and granuloma formation. This poses a threat for reactivation or dissemination of infections, particularly atypical bacterial, mycobacterial, fungal, and viral infections [17].

According to the World Health Organization, nearly one-third of the population has asymptomatic or latent tuberculosis. Less than 10% of these latent tuberculosis cases reactivate, but these cases account for nearly 80% of active tuberculosis cases. The overall incidence of tuberculosis is decreasing worldwide, but it remains a concern in patients receiving biologics such as TNF-*α* inhibitors, interleukin antagonists, and JAK inhibitors.

Eight cases of TB after ruxolitinib use in patients have been previously reported in literature (Table 1) [5–7, 9–12, 18]. Dissemination of TB was reported in five of these cases [5, 7, 9, 10, 12], while the remaining two cases reported were of reactivation pulmonary TB [6] and extrapulmonary TB [11]. Therapy with ruxolitinib was withheld, and standard four-drug ATT was given in all eight cases except one [5]. Due to a relapse of MF syndromes, ruxolitinib therapy was reinitiated with success by Palandri et al. and Hopman et al. [9, 11]. Duration of treatment varied from 6 months [11] to 12 months in cases with disseminated TB [5, 9, 12]. At the completion of standard ATT, Palandri et al. chose to maintain long-term prophylaxis with isoniazid with no evidence of subsequent TB reactivation [11]. More recently, Branco et al. recently described a case of disseminated TB, occurring in a ruxolitinib treated patient, where ruxolitinib therapy was maintained while patient received rifampin [5].

Based on our experience, before initiating treatment with ruxolitinib, we recommend TB screening with MTB QuantiFERON test especially for patients from TB endemic areas or with prior history of TB exposure. If latent TB is diagnosed, treatment for latent TB per Infectious Disease Society of America guidelines should be considered before starting treatment with ruxolitinib [19].

## Figures and Tables

**Table 1:** Summary of cases of *Mycobacterium tuberculosis* after receipt of ruxolitinib described in the literature.

	Case 1	Case 2	Case 3	Case 4	Case 5	Case 6	Case 7
Age (y)/sex	78/F	78/F	72/M	68/M	82/M	65/F	62/M

Infection	Disseminated TB	Disseminated TB	Miliary TB	Pulmonary TB	Reactivated pulmonary TB	Extrapulmonary TB	Disseminated TB

Timing of infection after start of ruxolitinib	1.5 years	Unspecified	5 months	4 weeks	2 months	4 months	7 weeks

Treatment of infection	ATT	ATT	ATT	ATT	ATT	ATT	ATT

Resolution of infection after treatment		Yes	No	No	Yes	Yes	Yes

Ruxolitinib therapy after diagnosis of infection	Continued	Discontinued at diagnosis of infection	Discontinued	Discontinued	Discontinued	Discontinued	Discontinued

Reintroduction of ruxolitinib during treatment of infection	Ruxolitinib continued without interruption	No	No	No	No	Restarted after 6 months of ATT	Restarted

Ruxolitinib resumed after completion of infection treatment	Continued without interruption	Unspecified	No	No	Unspecified	Ruxolitinib continued with isoniazid prophylaxis	Continued

Relapse of infection	No	No	N/A	N/A	No	No	No

Outcome	Alive	Alive	Died	Died	Alive	Alive	Alive

Year/reference	2016/[5]	2015/[12]	2015/[10]	2015/[10]	2015/[6]	2015/[11]	2014/[9]
